# Spatio-temporal interactions between the red fox and the wolf in two contrasting European landscapes

**DOI:** 10.1038/s41598-023-50447-z

**Published:** 2024-01-02

**Authors:** Lorenzo Lazzeri, F. Ferretti, M. Churski, T. A. Diserens, R. Oliveira, K. Schmidt, D. P. J. Kuijper

**Affiliations:** 1 Research Unit of Behavioural Ecology, Ethology and Wildlife Management, Department of Life Sciences, University of Siena, Via P.A. Mattioli 4, 53100 Siena, Italy; 2 NBFC, National Biodiversity Future Center, 90133 Palermo, Italy; 3 Mammal Research Institute, Polish Academy of Sciences, Stoczek 1, 17-230 Białowieża, Poland; 4 Faculty of Biology, University of Warsaw, Miecznikowa 1, 02‑097 Warsaw, Poland

**Keywords:** Ecology, Zoology, Ecology

## Abstract

Relationships among carnivore species are complex, potentially switching from competition to facilitation on a context-dependent basis. Negative associations are predicted to increase with latitude, due to limited resources emphasising competition and/or intra-guild predation. Accordingly, a stronger negative correlation between large- and meso-carnivore abundances should be expected at higher latitudes, with a substantial spatio-temporal partitioning favouring interspecific coexistence. Human presence may influence spatio-temporal relationships between (meso)carnivore species, as it can be perceived as a risk factor, but anthropogenic food can also provide an important additional food resource. Using camera-trap data, we studied the spatio-temporal associations between two of the most widespread carnivores in Europe, i.e., the red fox and wolf. We compared their monthly/daily spatio-temporal partitioning between two different landscapes: Białowieża Primeval Forest (Poland) and the Mediterranean Maremma Regional Park (Italy). We predicted a stronger interspecific partitioning, as well as more attraction of red foxes to humans in the northern site (Poland). Temporal activity patterns of the two carnivores overlapped in both sites, and their detection rates were positively associated, even though in weaker way in Poland. We observed a positive spatial association of red foxes with human activity in Białowieża, but not in Maremma. This association occurred only at a monthly temporal scale and disappeared at a daily scale, suggesting some disturbance in the shorter term. Our results provided partial support to our predictions and suggest that, despite the ecological differences between our study areas, only weak differences in wolf-fox relations were observed, suggesting that red fox responses to wolves may be relatively comparable over large spatial scales.

## Introduction

Large, apex carnivores can suppress mesocarnivore numbers^1–3^. As the presence of larger carnivores poses a lethal threat to most mesocarnivores, pronounced behavioural impacts on the latter can be expected. However, relationships between large carnivores and mesocarnivores are complex, often being influenced by several factors such as prey availability, predator density, presence of other potential competitors and, potentially, humans^4–7^. Moreover, interspecific relationships in carnivore communities can range from facilitation (e.g., via carcass provisioning) to competition or a combination of both, leading to direct killing or behavioural suppression^1,6, 8^. Nevertheless, carnivores can limit the effects of interspecific competition through resource partitioning along spatial, temporal and/or dietary axes^9–11^. Mechanisms leading to interspecific coexistence through ecological partitioning may act at different spatial and/or temporal scales^7,12^. Accordingly, there is growing evidence that this partitioning between carnivores acts especially at finer temporal scales, such as on a daily basis or even shorter time scales^13,14^.

Interactions among carnivore species may trigger effects across multiple trophic levels^4^. Understanding these interactions between carnivore species is important especially for European ecosystems recently recolonised by large carnivores. In Europe, the recovery of large carnivores is a recent process^4,15^ and information on the impacts of large carnivores on mesocarnivores is still scarce and contradictory. Some studies indicate that mesocarnivores spatially avoid large carnivores^16^, but others did not observe avoidance^17,18^. European ecosystems, where different sized carnivores co-occur, are usually small in extent, have low heterogeneity of natural habitats and are often fragmented and under significant human pressure^4^. Humans can influence the behaviour of carnivores, affecting their interactions, with consequences in ecosystem functioning^4,19, 20^. Conversely, anthropogenic food can provide an important additional food resource for carnivore species^3,21^. As some species avoid human settlements more strongly than others, mesocarnivores can also profit from human presence by using it as a shield against larger carnivores^12,22^. Available studies strongly suggest that interactions between large and mesocarnivores are often context-dependent^4^. Thus, to increase our understanding of these interactions between apex predators and mesocarnivores, studies in different ecosystems are needed to determine the potential ecological consequences of the recovery of apex predators across European ecosystems.

A recent meta-analysis^6^ suggests a more negative correlation between large- and meso-carnivore abundances at higher latitudes, indicating stronger suppressive effects via both killing and fear effects. This review showed that (1) carrion density decreases with latitude; (2) use of carrion by mesocarnivores is the same or slightly increases with the latitude; (3) large- and meso- carnivore abundances become negatively correlated as latitude increases; (4) large- and meso-carnivore abundances are more negatively correlated with increasing size of study areas. Thus, the work by Prugh and Sivy^6^ suggested a strong role of latitudinal differences in influencing interspecific associations between carnivore species.

We built on the conclusions of Prugh and Sivy^6^ and studied the most widespread mesocarnivore in Europe, the red fox *Vulpes vulpes*, and the main apex predator of European ecosystems, the grey wolf *Canis lupus*. Foxes are among the major consumers of carcasses of wolf prey^23,24^, which may lead to a positive spatio-temporal association between these two species^17,25^. However, foxes can be killed by wolves, potentially leading to avoidance^1,24^, especially at finer spatio-temporal scales. Relationships between abundances of wolves and foxes have been shown to be highly variable ranging from negatively to positively correlated^6^, with no clear pattern emerging at a continental scale^26^. The different ways that large and mesocarnivores have been found to interact suggest that fox responses to wolf presence are complex and context-dependent. Moreover, red foxes and wolves may show different behavioural responses towards humans. The wolf generally avoids direct contact or interactions with humans and anthropogenic structures^27,28^. In contrast, the red fox is an opportunistic species that shows higher tolerance to human presence and can profit by exploiting human food subsidies^3,29^. The presence of wolves is therefore expected to affect fox spatio-temporal behaviour, with human activities potentially influencing their relationships.

We investigated whether the wolf could affect the spatio-temporal activity of foxes in two areas, in southern (Maremma Regional Park, Italy, *c.* 90 km^2^; latitude 42°; hereafter ‘Maremma RP’) and central-northern Europe (Białowieża Primeval Forest, Poland, *c.* 580 km^2^; latitude 52°; hereafter ‘Białowieża PF’), respectively. The southern, Mediterranean area is snow-free throughout the year (with temperatures that rarely drop below 0°) contrasted to which the northern one has a long winter with frequent snowfalls. The Maremma RP is much smaller and has higher human pressure (i.e., more widespread human presence), compared to the larger Białowieża PF. Moreover, in Maremma RP the wolf reappeared during last 2 decades, meanwhile in Białowieża PF it has been present for centuries. Our target carnivores are protected inside both areas.

Based on the large scale/global patterns showed in the meta-analyses by Prugh and Sivy^6^ and on the dissimilarities between our study areas, we predicted some potential differences between wolf and red fox spatio-temporal associations in Maremma RP and Białowieża PF. In Białowieża PF, the red deer is the primary prey species for the wolf followed by wild boar (in the years with high wild boar abundance). In Maremma RP, deer species and wild boar dominate the wolf diet, with anthropogenic food and livestock providing a negligible contribution to it (Maremma RP^17,30^). Carcasses of wolf prey are a substantial alternative food resources to foxes in the Mediterranean area, where ungulates have been reported to make up about 30% occurrences and 13% volume of fox diet^17^. In Białowieża PF foxes have been shown to kleptoparasitize 87% of wolf kills^23^, and ungulates were reported to make up 25% of the biomass in its diet^31^.

We predicted that red foxes in temperate Białowieża PF compared to Mediterranean Maremma will show (1) a lower temporal overlap, and/or (2) lower overlap in space with the wolf. Furthermore, we expected (3) a stronger avoidance by the red fox of the wolf at finer temporal scale (to avoid lethal outcomes) than at coarser ones (attraction to carcasses prevails). Lastly, we expected (4) a stronger positive association between fox activity, human activity, and settlements in Białowieża PF than in Maremma RP (to profit from direct and indirect food resources that human presence may guarantee).

## Materials and methods

### Study areas

**Figure 1:**
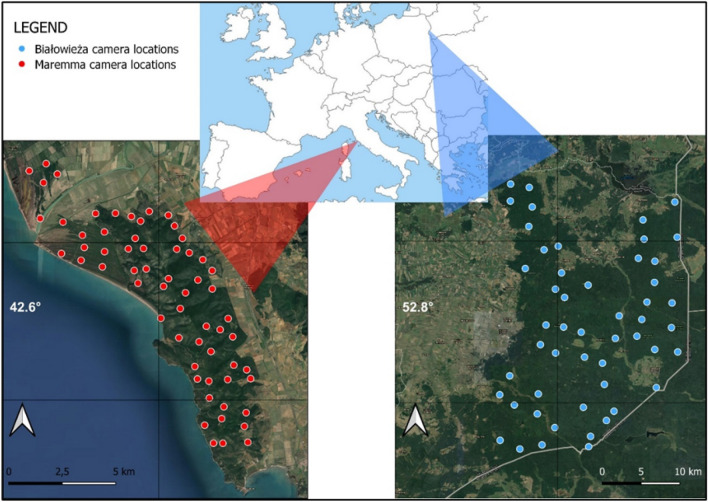
Satellite image of the Maremma Regional Park (left) and Białowieża Primeval Forest (right) with the camera locations highlighted. Maps created using the Free and Open Source QGIS 3.22.0 ‘Białowieża’ (https://www.qgis.org).

Our study areas were located (1) in a Mediterranean protected area in central Italy, Maremma Regional Park, (*c.* 90 km^2^; 42.633°, 11.092°), and (2) in Białowieża Primeval Forest in eastern Poland (*c.* 580 km^2^;52.732°, 23.868°), consisting of protected and managed parts (Fig. 1).

The climate in Maremma is typically Mediterranean with mild winters and hot summers, with a drought period in the summer months. The average annual temperature is *c.* 16°.

Such as most European ecosystems, Maremma Regional Park is an area that has been widely modified by humans over the centuries, and it currently has a diversified landscape characterised by hilly topography (maximum altitude: 417 m a.s.l.), Mediterranean forests and scrubwood with a succession of ecotonal and agricultural patches.

Maremma RP hosts a community of small-to-medium sized carnivores including the red fox, European badger *Meles meles*, wildcat *Felis silvestris*, stone marten *Martes foina* and pine marten *Martes martes*. The wolf returned to the Park permanently in the last decade after *c*. a century of absence^17^. During our study 2–3 wolf packs were reported^32^ Around 20–30 wild ungulates/km^2^ were estimated in summer (i.e., wild boar *Sus scrofa*: 10.5–15.1 individuals/km^2^; fallow deer *Dama dama*: 8.3–9.1 individuals/km^2^; roe deer *Capreolus capreolus*: 3.1–6.9 individuals/km^2^), on the last decade^30,33,34^.The Park Agency manages populations of fallow deer and wild boar through culling (both species) and trapping (wild boar), to limit the negative impacts of these ungulates on habitats/species with conservation relevance, and on agriculture. Livestock (*c.* 20 heads/km^2^) is also present, including free-ranging cattle and horses, as well as two sheep herds in sectors more marginal to the boundary to the Park^30^. The environment surrounding the Park is characterised by a mosaic of wooded areas, cultivated agricultural areas and extensive pastures where sheep farming is widespread; the largest city (Grosseto, 80,000 inhabitants) is located north of the Park, at a *c.* 15 km distance. No village occurs within the border of the Maremma RP. Five main small villages are located within 2 km from its limits and are inhabited by a total of 1710 people (http://italia.indettaglio.it/). Tourists visit the area mainly during the spring–summer, and their presence is concentrated especially around the beach; overall, an estimated *c.* 20,000–30,000 tickets are sold per year^35^. Motor vehicles are forbidden inside the Park, except on two roads; other forest roads are only used by authorized Park personnel, researchers, lumberjacks and farmers, in particular in the southern part.

Conversely, Białowieża PF is a relatively large temperate lowland forest with best-preserved natural character in Europe. The area is flat with an average altitude of 170 m a. s. l.. The climate is continental, summers are mild, and winters are often long, cold and snowy. Average annual temperatures are around 7°. The main forest association is oak-lime-hornbeam *Quercus robur, Tilia cordata, Carpinus betulus* with maple additives *Acer platanoides* and spruce *Picea abies* which grow on brown and podzolic soil. The wolf never disappeared from Białowieża PF for longer than 15–30 years during the last 200 years^36^, and 3–4 wolf packs were reported during our study period^37^. The Eurasian lynx *Lynx lynx* is also present (*c.* 15 individuals, Bubnicki et al. 2019)^37^, whereas the mesocarnivore community is diverse and comprises the red fox, raccoon dog *Nyctereutes procyonoides*, badger, pine marten and other mustelids. The ungulate density in Białowieża PF is estimated at *c*. 14 ungulates/km^2^ in summer (red deer *Cervus elaphus*: 6 individuals/km^2^; wild boar: 5.4 individuals/km^2^; roe deer: 2 individuals/km^2^)^37^. Wild boar numbers were strongly reduced after the African Swine fever outbreak and have stayed at low numbers ever since^38^. Two large species, the moose and European bison, are also present in Białowieża PF (moose: 0.08 individuals/km^2^^37^; bison: 1.2 individuals/km^2^: Białowieża PF unpublished data). Human density is low, including *c*. 13 people/km^2^ (it.db-city.com) and an estimated *c.* 200,000 tourists in Białowieża National Park concentrated around the Białowieża village (pers. comm. K. Niedzialkowski and W. Walankiewicz). There are only five paved roads of a total length of *c.* 50 km accessible to public cars in the Polish part of Białowieża PF.

### Camera-trap surveys

We collected data through intensive camera-trapping, with camera locations defined in sampling grids to cover entire study areas. In both areas, cameras were deployed on trails/ forest roads and animal trails as this increases the detection probability of carnivore species that preferentially use linear landscape structures^37^. Besides, camera trap surveys took place in both areas in late summer-autumn. During this period juvenile wolves begin to actively follow the adults in their activities and the wolf packs use their entire annual home range^39^. Therefore, it is one of the most suitable moments to evaluate the interactions between these two canids, as encounter rates between both species are predicted to be higher. To minimise the effects of climatic and biological differences between study areas, in the southernmost area (Maremma Regional Park) we postponed the study period by about a month compared to the northernmost area (Białowieża forest). In both sites, the cameras were generally put at 50–100 cm up to the ground, to allow the detection of both medium-sized and large carnivores and were spread across the entire landscape to cover the variation in environmental conditions that exist in the areas.

In Maremma RP, we used the data collected in October-December (autumn season) from 2017 to 2020. In 2017–2018 we worked in a c. 30 km^2^ sector of the study area in the northern Uccellina Mts. and pinewood (see for details^17,25^). Cameras were monthly rotated across 21 locations, covered during each 3-month period^25^. In 2019–2020 we extended the study area to cover a 60 km^2^ sector including all the Uccellina Mts., the pinewood, marshlands, and ecotone/open areas. In this period, 57 (2019) and 60 (2020) locations were monitored during the autumn season, by monthly rotating 19–20 cameras in order to monitor each location for about a month within the October–December period.

In Białowieża PF, we used data originating from a carnivore survey (see for a detailed description of the method^37^) that was conducted yearly between 2017 and 2020, as in Maremma RP in the 4 years the cameras were deployed between 15 August and late October/early November. The design consisted of 51–55 camera-trap locations (2017 and 2020) set along trails and forest roads to monitor large carnivores. In 2018 and 2019 we used the same locations along trails/forest roads, and additionally we deployed one extra camera at each point in the forest, approximately a hundred meters from the road (*N* = 98–100 camera locations in total).

### Spatial relationships between the red fox and wolf

For each location, we estimated the detection rates of both the red fox and the wolf as the ratio of the number of independent events over the number of camera-trap days. When the same camera-trap took more than one video of the same species within less than 30 min, we counted them as one event^40–42^. We also considered the rate of people activity, to evaluate its potential effects on spatial patterns of our focal species. We classified as “People” the following categories: runners, bikers, hikers, field operators, and vehicles with motor and without. In the case of detections of people, we considered a three-minute threshold between consecutive detections, when we were able to assess that they belonged to different people/groups^43^.

Whether and how species interact may depend on the temporal scale of the analysis. For example, foxes may avoid locations that have overall (on a large time-scale) higher visitation rates by wolves. Alternatively, the interactions could take place at very fine temporal scales in which foxes only directly avoid wolves at locations with their recent presence. To include both these large and fine time-scales, we considered two temporal scales for analysing spatial relationships between species. We defined a “coarser” temporal scale, i.e., the monthly detection rate of both species at each location, corresponding to the duration of each camera-trap deployment (i.e., c. 30–50 days). Besides we analysed a finer temporal scale, i.e., the daily detection rate of both species to check the potential for spatial associations during the same sampling days.

### Spatial relationships at a coarse (monthly) temporal scale

For the monthly scale relationships, we used generalized linear mixed models (GLMMs) with negative binomial errors^44^ to evaluate the relationships between the spatial patterns of red fox, our focal species, and those of the wolf. We set our models in the R package ‘glmmTMB’^45^ and ‘lme4’^46^. We built a full model for each study area. The number of detections of red fox in each camera-trapping location for each deployment was fitted as the response variable. In all models, the log (number of camera operating days) was included as an offset variable to standardize the response variables according to the actual sampling effort. We included the following predictors: (1) the detection rates of wolf; (2) people detection rate; (3) habitat (Maremma RP: oakwood; pinewood; shrub wood; ecotone/meadows; Białowieża PF: broad-leaved forest; coniferous forest; mixed forest; transitional woodland/shrub); (4) Distance from the nearest human settlement. We initially assessed whether to include the lynx among predictors for Białowieża PF models, considering its potential role as a competitor for the fox^26,47^. However, the lynx has a low density in Białowieża PF^37^, which is supported by the extremely small number of detections collected throughout our study (only 43 detections in 4 years). Hence, we decided to use only the wolf even to a better comparison between the areas’ models. In this study we put as predictor the variable ‘habitat’ on the camera location to control for potential effects of habitat type on the detection rate of our focal species, that can change in relation to different habitat. The habitat in each camera-trapping site was assessed through local land use maps (Maremma RP: see^48^; Białowieża PF: clc.gios.gov.pl). The distance from the nearest human settlement was estimated in QGIS, by using the layer of land use and cover 2018 Corine Land Cover of Italy and Poland in which we included as ‘permanent human settlements: (a) continuous and discontinuous urban structures (with at least ten houses)^27^; (b) industrial and commercial units. Data on people detection rates were not available for 2018 in Maremma RP, because people detections were not reported during this period. Thus, for Maremma RP, we conducted two analyses: a first one, considering only the years when people data were available and including all the above-mentioned predictors, and a second one, considering all the four study years, and where we did not include people detection rate among predictors. Since the results pointed in the same direction with both two approaches, in the main text we show only the results of models built through the datasets without the year 2018 that includes the people detections (for models with full dataset, see Supplementary Materials 1S–2S). We used the ‘Year’ as the random effect to account for potential differences between years. Absence of collinearity among linear predictors was checked preliminarily: pairs of predictors included in models did not show correlation coefficients >|0.6|^49^.

Subsequently, all possible models were calculated for each study area, including all possible combinations of considered predictors, since all of them reflected different a priori hypotheses, and were evaluated through a model selection procedure based on a comparison of AICc scores (Akaike Information Criterion). We used the model selection with the nesting rule to avoid retaining overly complex models^50,51^. We identified the best model as the most parsimonious one, i.e., the one having the lowest AICc^50,52^. Moreover, we selected for inference all models with AICc ≤ 2^51,52^, and among these, those which were not more complex versions of any simpler model^52^. Model selection was conducted using the R package ‘MuMIn’^53^. We estimated the parameters (95% confidence intervals and *B* coefficients, which is the degree of change in the response variable for every 1-unit of change in the predictor variable) of the best model by using the R package ‘lme4’^46^. Then, the best model was validated by visual inspection of the distribution of residuals^44^ through the ‘DHARMa’ package^54^. Model weight was standardized within the subset of selected models.

### Spatial relationships at fine (daily) temporal scale

The two study species showed consistently nocturnal temporal patterns (see “Results” and^17^). We categorised the sampling to avoid that detections of each species occurring on the same night could be treated as events relevant to different sampling days (e.g., events obtained at 23:50 and 00:05). Thus, we reframed the datasets by considering 12:00 (midday) as starting time for each day. We used the same structure of models as described above for the “coarse scale” but, as a response variable, we used the number of fox detections for each day in each camera location. These response variables were modelled through a zero-inflated negative binomial error distribution. The numbers of daily detections of wolves and people were considered as predictors, together with habitat and distance from human settlements. We put location of cameras and year as random effects.

### Temporal relationships

We used R 4.2.0 in RStudio version 4.0.3^55,56^ to estimate temporal activity patterns of our focal species (Maremma RP: red fox, wolf; Białowieża PF: red fox, wolf), as well as people, using the non-parametric kernel density estimation^57^. To ensure that our observations were independent we used the same approach described above (see “Spatial relationships between the red fox and wolf”). Then, we also estimated 95% confidence intervals of activity patterns as percentile intervals from 1000 bootstrap samples^58^. We used package ‘overlap’^57^ to evaluate the temporal overlap between each pair of species (e.g., wolf-red fox; red fox- people) in each study area both pooling data across years, and for each study year separately. We calculated the Δ_4_ estimator coefficient when the smallest sample of each pairwise comparison was ≥ 75 events, and Δ_1_ when the sample was < 75^57,59^. Overlap was defined as “low” when it was < 0.50, “moderate” when included between 0.50 ≤ Δ ≤ 0.75, “high” with Δ > 0.75, according to Monterroso et al.^60^. We calculated the 95% confidence intervals for overlap coefficients as percentile intervals from 1000 bootstrap samples^57^. Then, we compared the distribution of temporal activity between species through the Watson’s two-sample test of homogeneity, to test the uniformity of the two pair distributions^61^.

### Spatio-temporal partitioning (time to encounter)

For this analysis we measured the temporal distance, per camera location, between the two target species, the wolf and red fox. We considered only the direct detection of our target species, where in between did not pass other animals, in order to minimise the possible effect of other species on the relation assessment. We took the temporal distance between "pairs" of species into account when the wolf passed first and the red fox after, while "pairs" in the opposite order were used as control. We set a GLMM (gaussian family), and we put the log-transformed time difference between the two passages as response variable, the "pair" (wolf-red fox; red fox-wolf) as predictor, and "year" as a random effect^62^). When the beta is negative, it suggests there is no avoidance.

## Results

In Maremma RP, we obtained 1473 fox and 722 wolf detections over 3726 camera days for the full dataset. When the dataset without the year 2018 was considered, we obtained 1233 red fox, 605 wolf and 2236 people detections in 3372 camera days. In Białowieża PF, we obtained 2057 red fox, 618 wolf, and 9234 people detections, in 12,739 camera days.

### Spatial relationships

**Figure 2:**
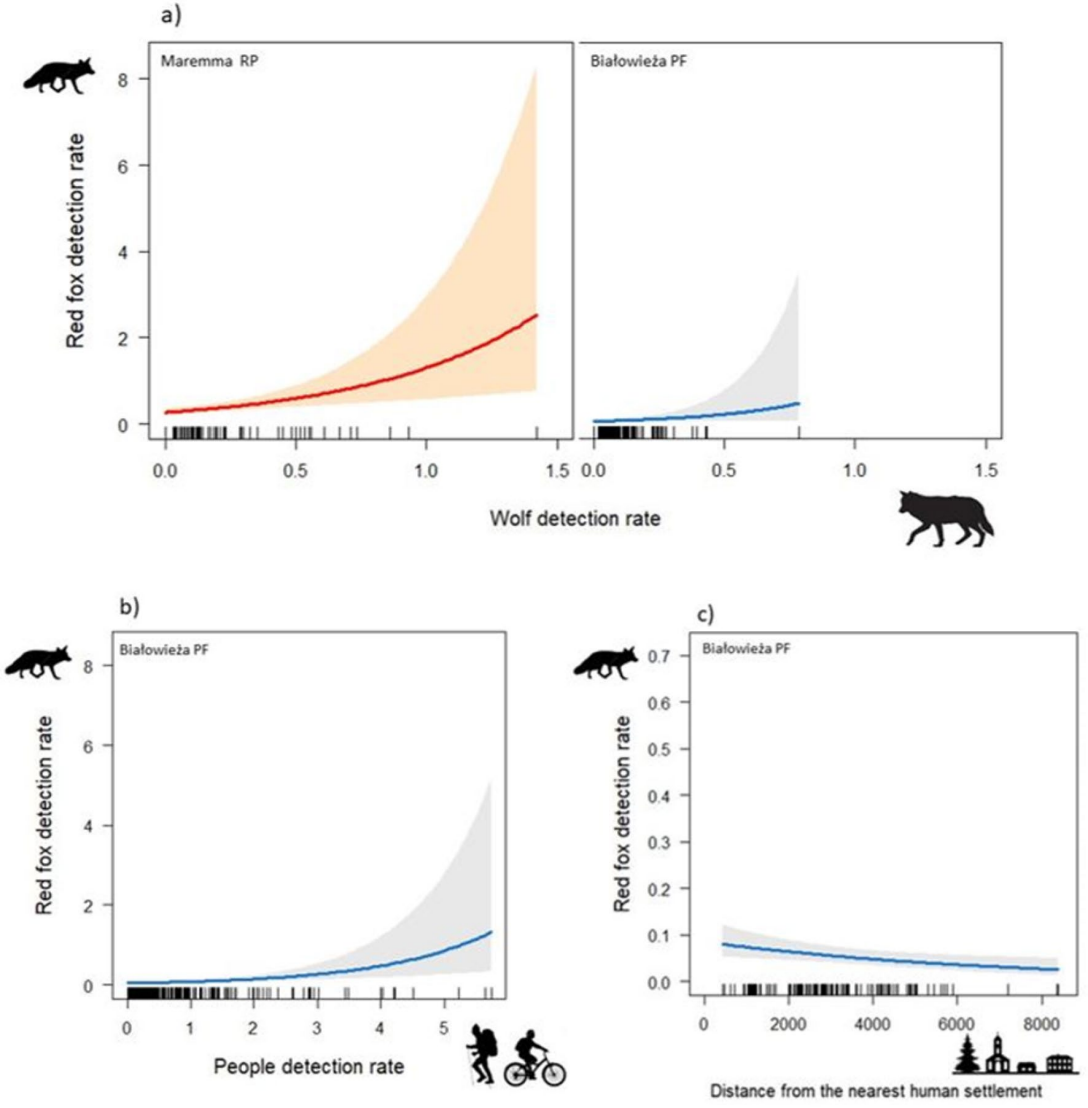
Relationships between monthly red fox and wolf detection rates in Maremma RP and Białowieża PF (**a**) and people detection rates (i.e., number of detections per working days in each camera locations) (**b**) and distance to the nearest human settlement and (**c**) in Białowieża PF. Relationship with anthropogenic factors in Maremma RP is not shown due to its insignificance. The coloured lines (red for Maremma RP and blue for Białowieża PF) indicate the estimated relationships; the grey and pink shaded area indicated 0.95 confidence intervals of the relationships. The distribution of data points is indicated at the x-axes by rugs (indicate partial residuals).

**Figure 3:**
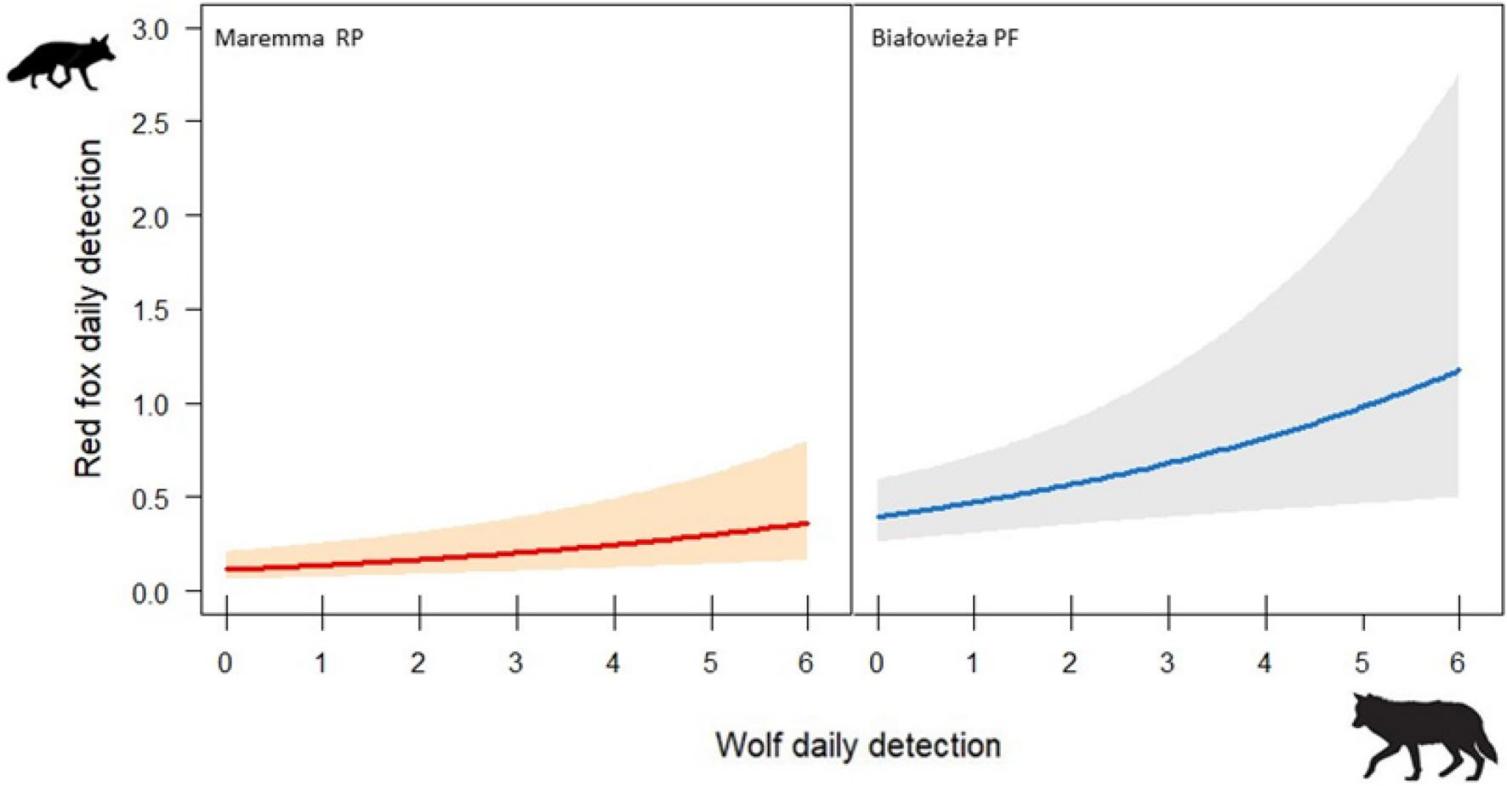
Relationships between daily red fox and wolf detection dates (i.e., number of detections each day at each camera location) in Maremma RP and Białowieża PF. Blue and red lines indicate the estimated relationships; the grey and pink shaded area indicated 0.95 confidence intervals of the relationships. The distribution of data points is indicated at the x-axes by rugs (indicate partial residuals).

In Maremma, at the coarse temporal scale, two models were selected for assessing spatial variation in fox detection rates (Table 1). Wolf detection rates were included in both models and were positively associated with fox detection rates (Table 2; Fig. 2). The predictor ‘distance from nearest human settlement’ was included in the best model, but its relationship with fox detection rate was not statistically supported (Table 2; Fig. 2). At a finer temporal scale, only the best model was selected, including the positive effect of wolf daily detection rates (Tables 1, 2; Fig. 3).

**Table 1:** Results of model selection for factors influencing spatial variation of red fox detection rates in Maremma RP and Białowieża PF at different temporal scales (coarse: *c.* 1 month; fine: daily) estimated through generalized linear mixed models with negative binomial errors.

Study area	Response variable	Model	Variables	K	logLik	AICc	ΔAICc	Weight
Maremma RP	Red fox—coarse	**Best**	**Wolf + distance human settlements**	**5**	**− 400.312**	**811.1**	**0.00**	**0.341**
		**Second**	**Wolf**	**4**	**− 401.401**	**811.1**	**0.01**	**0.340**
		Third	Wolf + people + distance human settlement	6	− 400.033	812.8	1.65	0.150
		Fourth	Wolf + people	5	− 401.176	812.8	1.73	0.144
		Fifth	Wolf + habitat + distance human settlements	8	− 399.568	816.4	5.24	0.025
	Red fox—fine	**Best**	**Wolf**	**6**	**− 2302.862**	**4617.8**	**0.00**	**0.493**
		Second	Wolf + people	7	− 2302.743	4619.5	1.77	0.203
		Third	Wolf + distance human settlements	7	− 2302.781	4619.6	1.85	0.196
		Fourth	Wolf + people + distance human settlements	8	− 2302.664	4621.4	3.62	0.080
		Fifth	Wolf + habitat	9	− 2302.705	4623.5	5.72	0.028
Białowieża PF	Red fox—coarse	**Best**	**Wolf + people + distance human settlements**	**6**	**− 830.976**	**1674.2**	**0.00**	**0.571**
		Second	Wolf + people + habitat + distance human settlements	9	− 829.048	1676.7	2.47	0.166
		Third	People + distance human settlement	5	− 833.287	1676.8	2.54	0.160
		Fourth	Wolf + people	5	− 834.104	1678.4	4.17	0.070
		Fifth	Wolf + people + habitat	8	− 831.730	1679.9	5.71	0.033
	Red fox—fine	**Best**	**Wolf + distance human settlements + people**	**8**	**− 4793.571**	**9603.2**	**0.00**	**0.384**
		**Second**	**Wolf + people**	**7**	**− 4794.736**	**9603.5**	**0.33**	**0.326**
		**Third**	**Wolf + distance human settlements**	**7**	**− 4795.418**	**9604.8**	**1.69**	**0.165**
		Fourth	Wolf	6	− 4796.607	9605.2	2.07	0.101
		Fifth	Distance human settlements + people	7	− 4797.079	9608.2	5.01	0.024

The top-five models are shown, together with their number of parameters, AICc, ∆AICc and standardized weight. Selected models are shown in bold.

**Table 2:** Factors influencing spatial variation of red fox detection rates in Maremma RP and Białowieża PF at different temporal scales (coarse: *c.* 1 month; fine: daily) estimated through generalized linear mixed models with negative binomial errors.

Study area	Response variable	Model	Variables	*B*	SE	95% CIs
Maremma RP	Red fox—coarse	Best model	Intercept	− 1.074	0.129	[− 1.326, − 0.821]
			**Wolf**	**0.372**	**0.111**	**[0.154, 0.589]**
			Distance human settlements	0.154	0.104	[− 0.050, 0.357]
		Second model	Intercept	− 1.065	0.123	[− 1.306, − 0.824]
			**Wolf**	**0.316**	**0.106**	**[0.108, 0.524]**
	Red fox—fine	Best model	Intercept	− 1.396	0.169	[− 1.727, − 1.065]
			**Wolf**	**0.107**	**0.025**	**[0.057, 0.156]**
Białowieża PF	Red fox—coarse	Best model	Intercept	− 1.984	0.218	[− 2.412, − 1.557]
			**Wolf**	**0.243**	**0.119**	**[0.009, 0.476]**
			**People**	**0.551**	**0.122**	**[0.311, 0.791]**
			**Distance human settlements**	**− 0.207**	**0.081**	**[− 0.364, − 0.049]**
	Red fox – fine	Best model	Intercept	− 2.690	0.307	[− 3.292, − 2.089]
			**Wolf**	**0.052**	**0.019**	**[0.015, 0.089]**
			People	− 0.048	0.026	[− 0.099, 0.002]
			Distance human settlements	− 0.183	0.120	[− 0.417, 0.052]
		Second model	Intercept	− 2.694	0.307	[− 3.296, − 2.092]
			**Wolf**	**0.052**	**0.019**	**[ 0.015, 0.088]**
			People	− 0.048	0.026	[− 0.099, 0.002]
		Third model	Intercept	− 2.683	0.306	[− 3.282, − 2.083]
			**Wolf**	**0.052**	**0.019**	**[ 0.015, 0.089]**
			Distance human settlements	− 0.182	0.118	[− 0.414, 0.049]

Estimates of model coefficients (*B*), their standard errors (SE) and 95% confidence intervals (95% CIs) and *p *values of selected models are shown. In bold, predictors for which an effect on fox detection rates was statistically supported.

In Białowieża PF, at the coarse temporal scale, only the best model for fox detection rates was selected (Table 1). A positive association was supported between fox and wolf detection rates (Table 2; Fig. 2). Fox detection rates increased with people detection rates and decreased with increasing distance from human settlements (Table 2; Fig. 2).

At the finer temporal scale, three models were selected and supported the positive effect of wolf daily detection rate; conversely, the effects of distance from human settlements and people daily detection rates were not statistically supported (Tables 1, 2; Fig. 3).

### Temporal relationships

**Figure 4:**
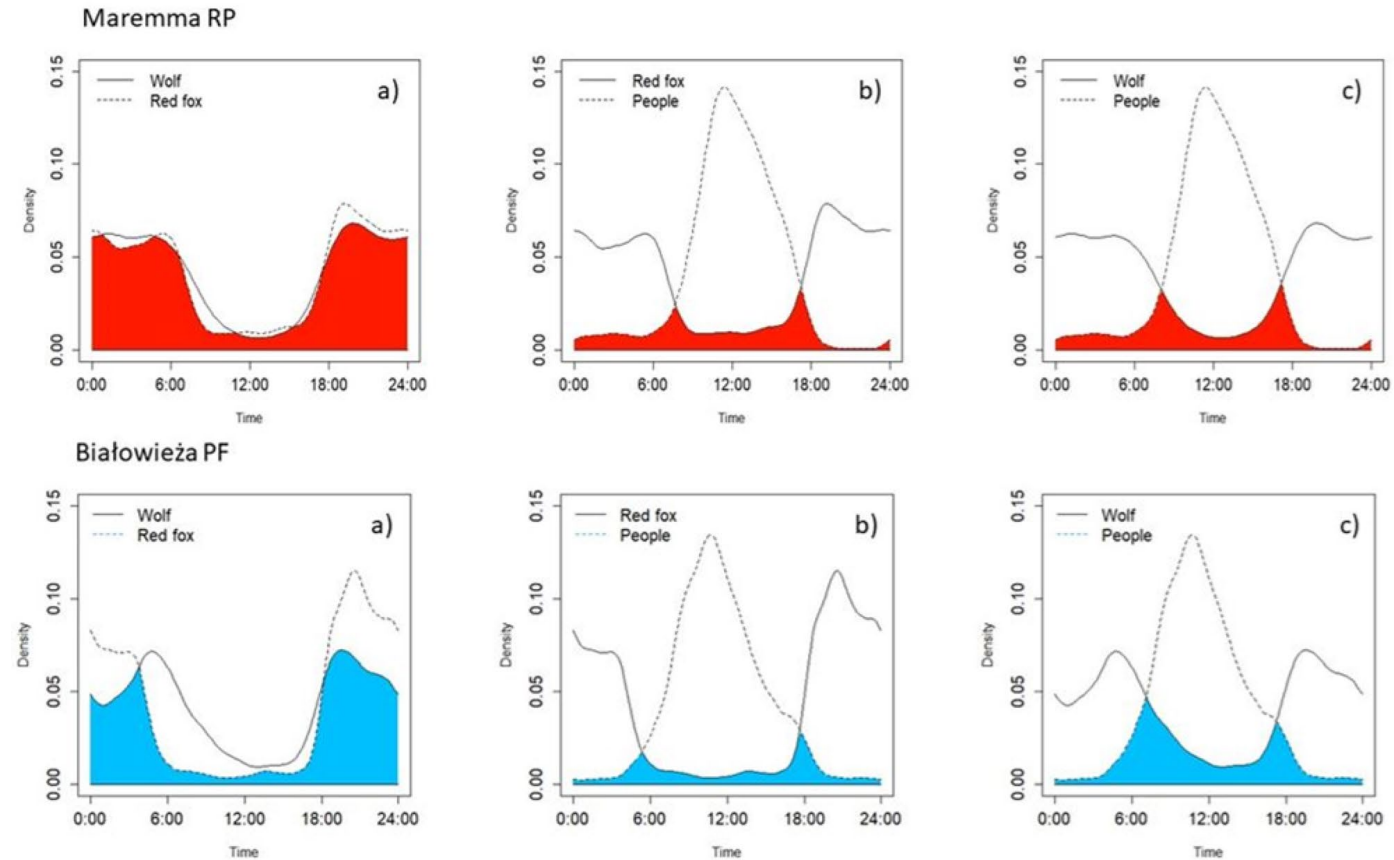
Temporal overlap between red fox and wolf (**a**); between red fox and people (**b**); and between wolf and people (**c**) in late summer-autumn 2017–2020, in the Maremma RP and Białowieża PF. The coloured sector indicates the area of overlap (red: Maremma RP; blue: Białowieża PF).

In Maremma RP, the red fox had a ‘high’ (sensu^60^) overlap with wolf activity (Δ_4_ = 0.94; CIs = 0.91–0.97; Fig. 4a). No significant difference was supported between the activity patterns of the two species (Watson two-sample test W = 0.104, p > 0.05). Conversely, the overlap coefficient between the red fox and people activity was ‘low’ (Δ_4_ = 0.22; Cis 0.20–0.25; Fig. 4b), with temporal activity patterns of foxes and humans being significantly different (W = 46.266, p < 0.001). The overlap coefficient between the wolf and people activities was low (Δ_4_ = 0.24; CIs = 0.21–0.26 W = 28.594 p < 0.001) (Fig. 4c). Both carnivores had nocturnal and crepuscular behaviour, instead, the people had a peak of activity at midday.

In Białowieża PF, the red fox had ‘moderate’ (sensu^60^) overlap with wolf activity (Δ_4_ = 0.72; Cis 0.69–0.76; Fig. 4a), and the temporal distribution of its activity differed significantly from that of wolves (W = 4.292, p < 0.001). The overlap coefficient between the red fox and wolf *vs*. people patterns was low (for the fox: Δ_4_ = 0.16; Cis 0.14–0.17; W = 114.766, p < 0.001; for the wolf: Δ_4_ = 0.33; Cis 0.30–0.36; W = 28.696, p < 0.001) (Fig. 4b,c). Both carnivores had nocturnal and crepuscular behaviour, instead, the people had a peak of activity at midday (see Supplementary materials Fig. 1S).

In both study areas, the spatio-temporal analyses (time to encounter) did not provide support for significant differences between temporal distances of red fox detections after wolf detections and temporal distances of wolf detections after red fox at given camera-trap locations, wolf detections/presence were/was not related to the time to encounter foxes and vice versa (Table 3).

**Table 3:** Model selection for “time to encounter”, estimated through generalized linear mixed models in Maremma RP (N observations = 456, total period with 2018) and Białowieża PF (N observations = 209).

Study area	Variables	*B*	SE	95% Cis
Maremma RP	Intercept	− 1.582	0.169	[− 1.913, − 1.250]
	Pair (Wolf-Red fox)	− 0.033	0.183	[− 0.391, 0.325]
Białowieża PF	Intercept	− 1.143	0.162	[− 1.460, − 0.826]
	Pair (Wolf-Red fox)	− 0.262	0.216	[− 0.685, 0.160]

Effects of predictors included in the best model are shown: model coefficients (B), their standard error (SE), 0.95 confidence intervals (Cis), and p value (P).

## Discussion

We addressed spatio-temporal associations of the red fox with the major apex predator in Europe, the wolf, in two areas with different ecological contexts. Due to different latitudes, and dissimilar sizes of study areas (Maremma RP: *c.* 90 km^2^; Białowieża PF *c.* 580 km^2^), based on the large scale/global patterns shown in Prugh and Sivy^6^, we expected more negative interactions between carnivores, as well as a greater attraction of foxes to humans, in Białowieża PF than in Maremma RP, leading to a greater spatio-temporal partitioning between the fox and the wolf in the former area than in the latter one. However, the results only partially supported our predictions. Although the spatio-temporal overlap between the red fox and wolf was lower in Białowieża PF than in Maremma RP, we detected no clear evidence for avoidance. In contrast, our results overall support a lack of direct negative association between the two carnivores in both areas, suggesting that positive interspecific associations may be more common than other studies predicted^1–3^. Furthermore, fox detection rates were associated positively with anthropogenic features and people detections only in Białowieża PF. However, this association emerged only at the monthly scale, but not at the daily scale, suggesting some avoidance may occur at the finer temporal scale.

In both our study areas, wolves and foxes had strictly nocturnal/crepuscular activity patterns, with some peaks of activity at dawn/dusk, in line with previous studies^63,64^. Nocturnality has frequently been interpreted as a general response of wild animals to human activity, which is usually concentrated during daylight hours^65,66^ and has been regularly observed in our study species. However, temporal activity patterns of the fox were significantly different from those of the wolf in Białowieża PF but not in Maremma RF (Fig. 1S). Our results for Maremma RP confirm previous observations that foxes show a remarkable temporal overlap with the wolf^17,32^, with a greater synchronisation at sites more used by wolves than at sites less used by this apex predator^25^. Our Białowieża PF findings agree with results obtained by Haswell et al.^12^ in another European area hosting the same carnivore community (Plitivice Lakes National Park, Croatia). Furthermore, at neither of our study areas did we find a negative spatial association between fox and wolf activity (see also^32^), at either the coarser or finer temporal scale. In contrast, we found a positive spatial association of the activity of the two carnivores, especially at the fine (daily) time scale. Thus, we found no support for temporal or spatial avoidance of the wolf by the red fox, and our results suggested that foxes did not avoid places recently visited by wolves.

Recently, Prugh and Sivy^6^ reported that the potential for negative *vs.* positive associations between apex predators and mesocarnivores increases with latitude and the size of study sites. Although our results suggest a greater overlap between foxes and wolves in the southernmost site than in the northernmost one, no strong avoidance was observed. Sivy et al.^5^ observed guild-wide negative responses to wolves in their study areas in Alaska. Although the local carnivore community included also intermediate carnivores such as the wolverine *Gulo gulo* and the coyote *Canis latrans*, a potential negative effect was recorded also on smaller carnivores i.e., red foxes and martens. These authors suggested that co-occurrence of small carnivores in the vicinity of carcasses could elicit a generalized predatory response from wolves when present^5^. Accordingly, their results suggested that the attraction to carcasses may result in positive local-scale associations among carnivores, but scavenging-related mortality could lead to negative landscape-scale effects of apex predators (“*fatal attraction hypothesis*”, and^5^). Similarly, negative numerical correlations between fox density and wolf pack size were observed in Scandinavia at the scale of wolf pack territory, although potentially mediated by habitat and resource availability^67^. Both our study areas are more productive and with less hostile climates than Alaskan and Scandinavian ones, and intermediate carnivores are absent. Moreover, compared to many boreal ecosystems, Maremma RP and Białowieża PF, host relatively high ungulate densities (respectively 20–30 ungulates/km^2^ and 14 ungulates/km^2^), which could explain the rather subtle differences we found in wolf-fox interactions between both areas. In our study areas, wolf food habits have been shown to be dominated by large, wild prey (Białowieża PF:^68^; Maremma RP:^17,30, 32^, while foxes are significant scavengers of wolf kills (Białowieża PF:^23^; Maremma RP:^17^. A concurrent study in Maremma reported a three-to-fourfold increase in the occurrence of ungulates in the fox diet with respect to times when the wolf did not present in the area^17^; (see also^69^, for fox-Eurasian lynx interactions): this may be evidence for trophic facilitation mediated-by an increase of foraging opportunities provided through leftovers. Furthermore, a study based of 3 whole-year data found no support for spatio-temporal avoidance^32^. Our results suggest that wolf movements might influence those of red foxes, possibly indicating a predominance of the attractive component, at least at the scales we analysed. Carcasses left by the wolf can become a hotspot for scavengers and sometimes trigger an apex predator reaction (e.g., disturbance or killing), but this last behaviour could depend on the intensity of total carcass biomass consumed by foxes (i.e., scavenging) and resource overlap^5,7^. Moreover, our results suggest that fear of wolves by foxes may be not severe, leading to lack of avoidance at spatial and temporal scales. Suggestively, (1) intraguild predation within Carnivora is less common among species pairs that are either very different or similar in size^1,8^, and (2) in our study areas, smaller carnivores such as foxes have been reported as being only negligible components of wolf kills^68^ or diet^32^. Nevertheless, our results may also suggest that the lack of support to strong spatio-temporal avoidance is associated with higher costs for individual foxes of missing foraging opportunities in relation to the actual risk of being killed by a wolf.

Overall attractive rather than avoidance relations seemed to prevail, although data on fox mortality and population dynamics would be needed to test for the effects of wolves on fox populations^7^. Future work should consider potential mechanisms of spatio-temporal partitioning acting at finer spatial scales^13,70^ through satellite telemetry^7^ and/or multidimensional studies, as those potentially associated with the use of den refugia by foxes. These canids can use dens throughout the year and not limited to the period of birth and weaning of offspring^71^. The location and use of dens by foxes can also be influenced by the presence of predators^72^.

Anthropogenic influence can play a significant role in modulating interactions among carnivores^3,73^. Indeed, presence and activity of humans can impose synergistic risk factors to carnivores or generate the potential for attraction to anthropogenic food resources^3,4, 74^, ultimately influencing temporal and spatial patterns of carnivores^27,66, 75^. We predicted that human presence may affect the behaviour of an opportunistic mammal with synanthropic attitudes such as the red fox^3,76^, especially in the northernmost study area where competitive relations between wolf and fox were predicted to prevail. The results supported our expectations, with positive associations between red fox detection rates, human detection rates and human settlements occurring only in Białowieża PF, suggesting a more attractive role of human features to this opportunistic and ecologically flexible mesocarnivore than in Maremma. This could be related to foxes seeking shelter close to humans to avoid wolves (‘human shield effects’^77^), but looking at the overall positive relations we found between foxes and wolves, this seems an unlikely explanation. Alternatively, foxes could be present closer to human settlements in Białowieża PF because favourable foraging sites are present in the form of meadows near the villages. In these meadows rodents are abundant and therefore may attract foxes^31^. However, the relationship between people and fox detection rates switched to no effect at the daily temporal scale, suggesting that some avoidance could occur in the shorter term*.*

Our results suggest that some temporal partitioning between foxes and wolves may occur in Białowieża PF, although we found only minor differences between our study areas, with overall no negative relations potentially prevailing over competitive ones. In Europe, in the last decades, large predators are recolonising their historical ranges^4,15^. In Maremma, the re-occurrence and stable presence of wolves have been reported only in the last decade, after about a century of absence^17^. The great abundance of large ungulates has been suggested to make wolves relatively tolerant to foxes, thus reducing the potential for avoidance patterns to occur^17^. Our results suggest that even in the area with long-term wolf presence, foxes do not avoid wolves at the scales analysed. Both areas are characterised by relatively high ungulate densities and future work should assess how wolf-fox interactions may change in relation to changes in ungulate numbers.

Our work presents knowledge on the relationships between apex predators and mesocarnivores in European ecosystems, showing that positive relationships can occur rather than solely negative associations^1–3^. Although apex predators are often predicted to inspire avoidance in smaller carnivores, we did not find support for spatial or temporal negative association between the fox and the wolf in two contrasting study areas; rather, positive associations seemed to prevail. In turn, results suggest that the ecological differences between our study areas may not be large enough to elicit dissimilarities in wolf-fox relationships. Thus, apex predators (like the wolf) may not suppress the behaviour of smaller carnivores in all contexts^78^. Future studies may wish to explore under which contexts suppressive and facilitative effects occur.

### Supplementary Information

Supplementary Information.

## Data Availability

The datasets used and/or analysed during the current study are available from the corresponding author and FF (for Maremma RP) on reasonable request.
