# Supplementary material for: Spatio-temporal interactions between the red fox and the wolf in two contrasting European landscapes
Source: Sci Rep. 2024 Jan 2;14:221. doi: 10.1038/s41598-023-50447-z (PMC10762132; doi:10.1038/s41598-023-50447-z)
Supplement: Supplementary file 1 — Supplementary Information. [file 41598_2023_50447_MOESM1_ESM.docx]

Supplementary Materials

**Spatio-temporal partitioning between the red fox and wolf in two contrasting European landscapes**

**Table 1S**. Results of model selection (with 4 years including 2018, without “people” as predictor) for factors influencing spatial variation of red fox detection rates in Maremma RP at different temporal scales (coarse: *c.* one month; fine: daily) estimated through generalized linear mixed models with negative binomial errors. The top-five models are shown, together with their number of parameters, AICc, ∆AICc and standardized weight. Selected models are shown in bold.

| **Response variable** | **Model** | **Variables** | **K** | **logLik** | **AICc** | **ΔAICc** | **Weight** |
| --- | --- | --- | --- | --- | --- | --- | --- |
| Red fox - coarse | **Best** | **Wolf** | **4** | **-448.560** | **905.4** | **0.00** | **0.458** |
|  | Second | Wolf + Distance_human settlements | 5 | -447.768 | 906.0 | 0.57 | 0.345 |
|  | Third | Wolf + Habitat+ Distance_human settlement | 8 | -445.631 | 908.4 | 2.95 | 0.105 |
|  | Fourth | Wolf + Habitat | 7 | -446.912 | 908.7 | 3.26 | 0.090 |
|  | Fifth | Habitat | 6 | -451.771 | 916.2 | 10.76 | 0.002 |
| Red fox -fine | **Best** | **Wolf** | **6** | **-2639.190** | **5290.4** | **0.00** | **0.663** |
|  | Second | Wolf + Distance_human settlements | 7 | -2639.124 | 5292.3 | 1.88 | 0.259 |
|  | Third | Wolf + Habitat | 9 | -2638.694 | 5295.4 | 5.04 | 0.053 |
|  | Fourth | Wolf + Habitat + Distance_human settlements | 10 | -2638.488 | 5297.0 | 6.64 | 0.025 |
|  | Fifth | *null* | 5 | -2648.849 | 5307.7 | 17.31 | 0.000 |

**Table 2S** Factors influencing spatial variation of red fox detection rates in Maremma RP at different temporal scales (coarse: *c.* one month; fine: daily) estimated through generalized linear mixed models with negative binomial errors (with 4 years including 2018, without “people” as predictor). Estimates of model coefficients (*B*), their standard errors (SE) and 95% confidence intervals (95% CIs) and *P-*values of selected models are shown. In bold, predictors for which an effect on fox detection rates was statistically supported.

| **Response variable** | **Model** | **Variables** | ***B*** | **SE** | **95% CIs** |
| --- | --- | --- | --- | --- | --- |
| Red fox - coarse | Best model | Intercept | -0.979 | 0.134 | [-1.241, -0.717] |
|  |  | **Wolf** | **0.330** | **0.096** | **[0.143, 0.518]** |
| Red fox - fine | Best model | Intercept | -1.285 | 0.154 | [-1.587, -0.983] |
|  |  | **Wolf** | **0.102** | **0.023** | **[0.058, 0.146]** |

**
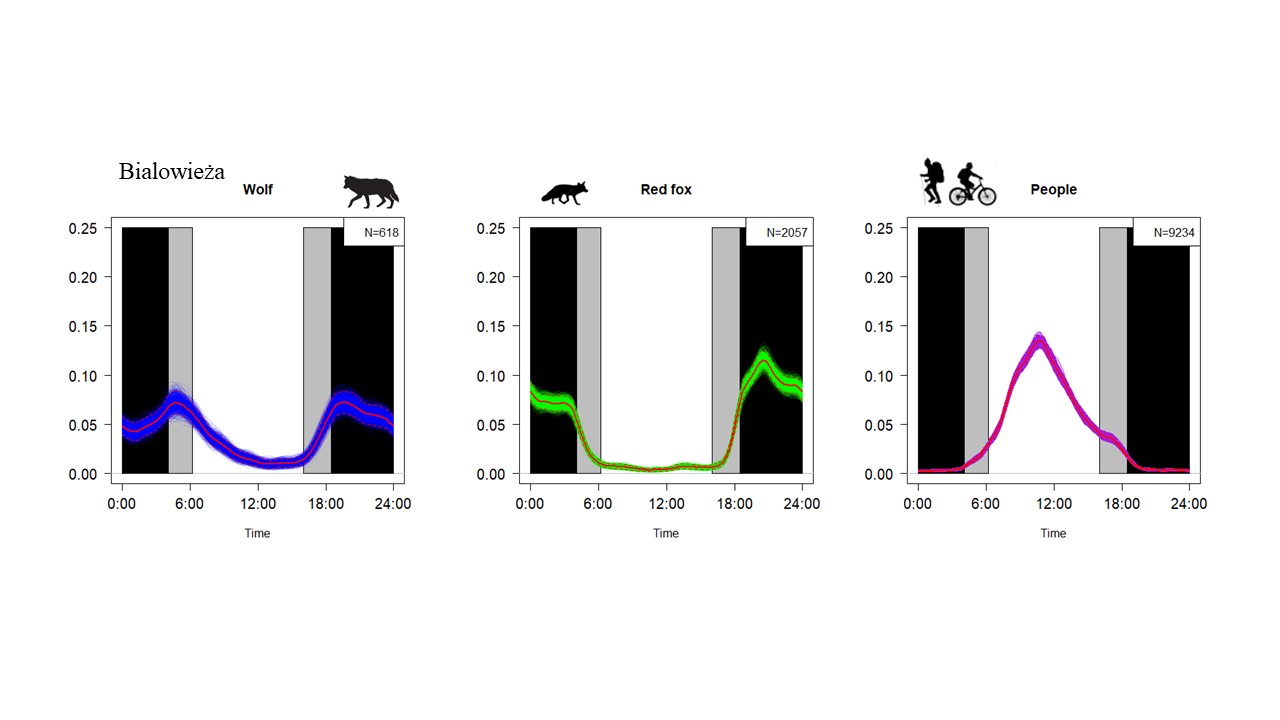

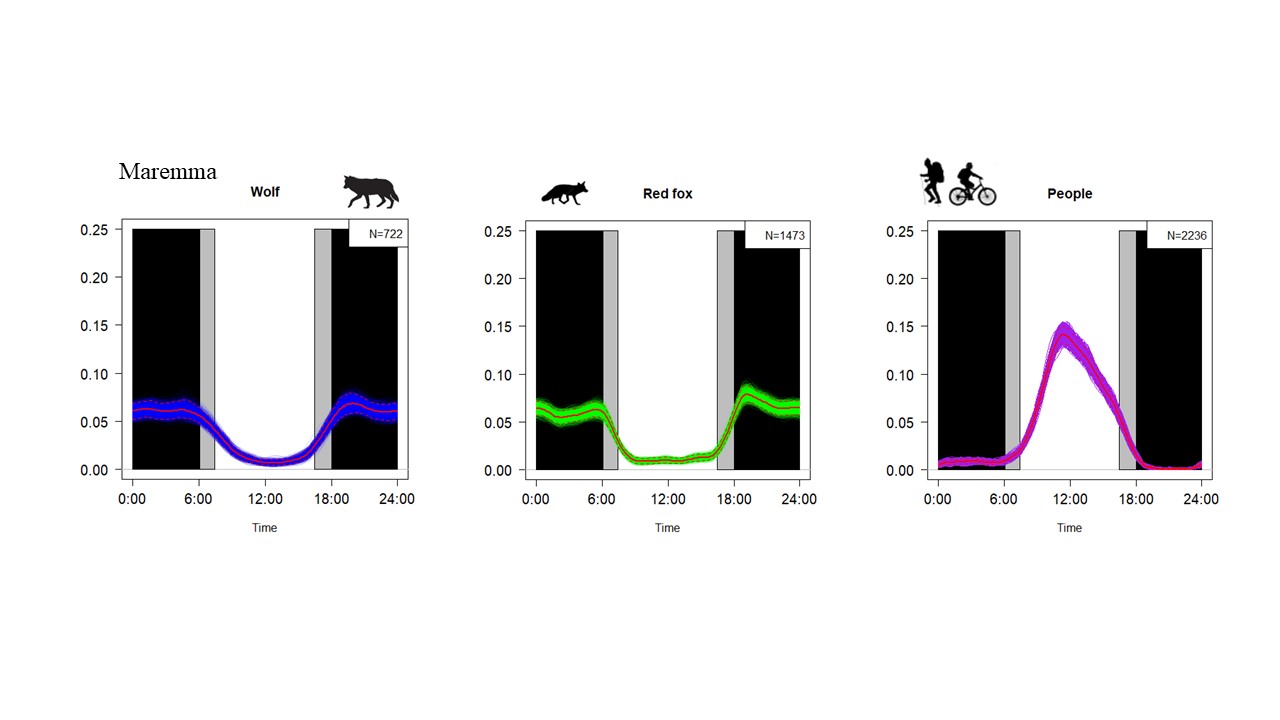
**

**Fig. 1S** Temporal activity patterns of wolf, red fox, and people in the Maremma Regional Park (2017-2020, Red fox and wolf activities also include the data of 2018) and Bialowieża Primeval Forest (2017-2020). Coloured lines represent bootstrapped estimates of activity patterns; dashed coloured areas represent 0.95 confidence intervals. Grey rectangles indicate dawn/dusk and black rectangles indicate the night. We represented the crepuscular time using the hour of dawn/dusk of our first and last day of sampling. Sample size is shown in boxes.
